# On the Present State of Medicine and Medical Education in Holland

**Published:** 1838-07

**Authors:** C. J. Nieuwenhuijs

**Affiliations:** Member of the Provincial Medical Commission for the Province of North Holland, &c. &c.


### PART FIFTH.

### Medical Intelligence.

---

### ON THE PRESENT STATE OF MEDICINE AND MEDICAL EDUCATION IN HOLLAND.

By C. J. Nieuwenhuijs, m.d., Member of the Provincial Medical Commission for the Province of North Holland, &c. &c.

### A. OF THE MEDICAL INSTITUTIONS.

Holland contains three universities,—those of Leyden, Utrecht, and Gröningen. Harderwyke and Franecker were formerly the seats of universities, but, owing to the small number of students at each, were disfranchised early in the present century. Linnæus for some time studied, and ultimately graduated, at Harderwyke; and here was published his Amænitates Academicæ. There are also three subsidiary colleges, or Athenæums,—one at Amsterdam, another at Franecker, and the third at Deventer. Each of these has five Faculties; and the medical Faculties are subjected to arrangements in accordance with the general system of instruction established in Holland. Wardens or directors (curators) are appointed to preside over each; and these functionaries are selected from the most respectable classes, and generally with reference to their literary acquirements. Every university has its rector-general, secretary, five deans and secretaries; one of each of the latter to each faculty. The rectorship is an annual office, held by the professors in rotation. The different faculties elect annually their own deans; but the secretaryships are for the most part life-offices, and are generally held by the senior professor: thus, Sandifort is secretary to the medical faculty in Leyden.

### I. THE UNIVERSITIES.

#### I. THE UNIVERSITY OF LEYDEN.

Among the universities, that of Leyden still holds the first place, on account of the excellency of its arrangements and institutions. It was founded in the year 1575, and contains the following professors: Of the Faculty of Physics and Natural History, nine; of the Faculty of Literature and Philosophy, eleven; of the Faculty of Medicine, five.

The following are the names of the professors, and the subjects of the lectures relating to medical science delivered in the Faculties of Physics, Natural History and Medicine, in the session of 1837-8:

C. G. C. Reinwardt Chemistry and Botany.
J. G. van Breda . Natural History, Anatomy, Physiology, and Comparative Anatomy.
J. van der Hoeven, (Prof. Extr.) Zoology, Comparative Anatomy and Physiology, Anthropology, Entomology.
A. H. van der Boon Mesch, (Prof. Extr.) Chemistry, Practical Chemistry, and Pharmacology.
G. Sandifort . . Anatomy, Physiology, Dissections.
M. J. Macquelyn . Pharmacology, Dietetics, Clinical Medicine.
J. C. Broers . . Surgery, Clinical Surgery, Midwifery, Forensic Medicine.
C. P. van der Hoeven, Pathology, Practice of Physic, Clinical Medicine, History of Medicine.

This university is well known, both on account of the distinguished men who have flourished in it, and for the excellence of its scientific museums and other arrangements. The Museum of Natural History is in no way inferior to that of Paris, and in many respects even excels it, especially in the beauty, variety, and extent of its osteological department. It has lately been augmented by a complete and systematically arranged collection of birds, which has been presented to it by the present director, Themmink. The Museum of Antiquities has been but recently established, and was not, in July 1837, open to the public: it is under the immediate superintendence of that learned antiquary, Dr. Lamans, and is augmenting daily both by purchases and presents. The anatomical and pathological collection, which is already very rich, is also constantly increasing, through the zealous perseverance of Professor Sandifort, who follows the track of his excellent father. The professor of anatomy is, *ex officio*, director of this collection. In it we find some of the most successful of Ruysch's injections, preparations of Albinus, father and son, Bonn, Rau, Brugmanns, Sandifort, sen., and others. The preparations from which the drawings were taken for Sandifort's large work, entitled "Observationes Anatomico-pathologicæ," are to be seen here. An extensive Japanese collection is under the direction of Dr. de Siebold, its principal collector, and is open to the public twice in the week. The University Library is extensive: it is very rich in old medical books and manuscripts, and is not behind any continental library in its collection of modern standard works of all languages. The Botanical Garden forms one of the great attractions of the town, of which the inhabitants may well be proud. It is situated at the back of the university, and extends from hence down to the Rhine, a branch of which entirely surrounds the town. The plants have all been arranged according to their natural order: this arrangement, with many other improvements, having been effected by the late and present directors, Brugmanns and Reinwardt. The lectures on botany are delivered in a theatre within the garden; and, on the conclusion of the lecture, the professor proceeds with the pupils to that division occupied by the natural family he has been discussing, and then enters somewhat more into the analogies and other particulars of the whole group: he is also ready to answer any enquiries that may be put to him by the pupils. The hothouses are large and extensive, and are well stored with oriental and other plants. A fine specimen of the Fraxinus ornus, planted by Boerhaave himself, when professor of botany and chemistry, is to be seen in the upper garden.

The lectures are (with the exception of the clinics) all delivered in Latin; some at the houses of the respective professors, others at the hospital: among the latter are medicine, surgery, midwifery, and the clinics.

The hospital is well arranged: it contains about fifty beds, nearly equally divided between medical and surgical cases, including among the latter midwifery; for it is also a lying-in establishment. The physicians are Professors Macquelyn and Pruijs van der Hoeven; the surgeon and obstetrician, Professor Broers. The pupils have the privilege of dressing and of prescribing for the patients, under the direction of the medical officers.

These are the principal of the public medical institutions. Our view of Leyden, as a seat of learning, would, however, be scarcely complete without referring to the private collection of Professor Broers, and to the magnificent library and museum of Professor Reinwardt. The specimens of diseased bone in the former may, from their beauty of preparation, their extent and rarity, be considered as unique. The latter collection is particularly rich in botanical works. The esteemed owners of them are always ready to make them as serviceable as possible. The emolument of the professors is derived from two sources, from government and from the pupils. Nothing but an adequate and extensive medical and surgical clinic is wanting to render the university complete.

When we consider that the expenses of the university amount yearly to the sum of 118,000 Dutch guilders,* (nearly 10,000*l.*) we cannot refrain from expressing our astonishment that it should be incomplete in any department, or leave anything to be wished for.

* A Dutch guilder, or florin, is in value about 1*s.* 8*d.* English.

### II. THE UNIVERSITY OF UTRECHT.

This university was founded in the year 1636, and celebrated, in June 1836, the jubilee of its two hundredth anniversary; of which a description has been published; and which will obviate the necessity of any further elucidation on our part.

In the year 1835, there were 519 pupils at this university.

The curators are six in number.

The professors of the Faculty of Physics and Natural History are seven in number, and of Medicine nine.

The following are the names of the medical professors, and the subjects taught by them:

T. L. C. Schroeder van der Kolk, Anatomy, Physiology, Pathological Anatomy, Dissections.
B. F. Suerman . . Pathology, Surgery.
J. T. Wolterbeck . Nature and Treatment of Diseases, General Therapeutics, Pharmacology, Semeiotics, Clinical Medicine, Clinical Midwifery.
N. C. de Fremery . Pharmacy (in the Dutch language), Chemistry, Mineralogy and Geology, Medical Police.
P. T. S. Fremery . Animal and Organic Chemistry.
C. A. Bergsma . . Medical and Economical Botany, Anatomy of Plants, Botanical Excursions.
T. Kops . . . . Botany, Physiology of Plants.
T. G. van der Ledth de Jeude, Natural History, Zoology, Comparative Anatomy.

Utrecht possesses a botanical garden, an anatomical theatre, and a physiological and pathological museum, which formerly belonged to Professor Bleuland, and was ceded by him to the university.

This university costs the government 70,000 florins yearly; notwithstanding which, it is but in a very incomplete state.

The Veterinary Medical Institution is unconnected with the university: it is situated outside of the gates of the town, is under the direction of Professor A. Numann, and is provided with a good hospital and other requisites for diseased animals. This school is said to have collected, by its own exertions, a very fine physiological and pathological cabinet; of which an account has lately been published.

### III. UNIVERSITY OF GRÖNINGEN.

This university is situated in the eastern part of Holland: it was founded in 1614. In the year 1835 it had only 276 students.

The curators are six in number.

The following are the professors of the Faculties of Medicine and Natural History:

S. E. Strating, J. Boast de la Faille, A. A. Sebastian, Physiology.
H. Wolthers . . Clinical Medicine, and Medicine.
W. Verschuir . . Clinical Surgery, and Surgery.
H. Jonxis . . . . Prosector of Anatomy.
J. C. Hoffman, S. Strating, Chemistry.
C. H. van Hall . Botany.

Although the university of Gröningen is of such little utility, its expenses are the same as those of Utrecht, namely, 70,000 florins a year. It is deficient in many requisites; the enumeration of which does not, however, belong to this place.

### II. SUBSIDIARY COLLEGES, OR ATHENÆUMS.

### I. ATHENÆUM OF AMSTERDAM.

This Athenæum was founded on the 8th of January, 1632, and has consequently existed 206 years. It is celebrated for the distinguished men that have adorned it; as a proof of which, we need only mention the names of Tulp, Ruysch, and A. Bonn in former times, and that of G. Vrolik in the present.

The curators are four in number.

The medical professors are: G. Vrolik, F. van der Breggen, W. Vrolik, W. H. Vriese, W. S. Swart, C. B. Tilanus, and G. C. B. Suringar.

These last two professors have been appointed honorary professors at the Athenæum, because they were professors at the Clinical School, an institution in some measure connected with the Athenæum, and which we shall therefore notice in this place, as well as all the other institutions in Amsterdam connected with medical science.

THE CLINICAL SCHOOL, HOSPITALS, &c.

By a royal decree, dated January 6, 1823, a so-called clinical school was established in the principal town of each province, for the education of surgeons, apothecaries, rural surgeons (Plattelands Heelmeesters), men-midwives (Vrowmeesters), and midwives (Vrowvrouwen). In accordance with this decree, there is now a clinical institution connected with the Athenæum, though the union between the two institutions is not so close as might be wished for the advancement of medical science. It was originally intended that the superintendence of the clinical school should be committed to the Medical Board in North Holland; but several circumstances, unconnected with this statistical account, have caused this intention to be modified, and at present the superintendence has been intrusted to the minister of the interior, who elects a board of commissioners, consisting of the governor of this province, or a member of the provincial States, as president, three members of the above-mentioned Medical Board, one member of the town-council, one curator of the Athenæum, and one director of the hospitals.

In this school the students are instructed in the following branches of science: Anatomy, Natural History, Physiology, Botany, Chemistry, Pharmacy, Materia Medica, Pathology, Therapeutics, Surgery, and Obstetrics. The instruction in all the Athenæums is conveyed in Dutch. Formerly the lectures at the Athenæum were delivered in Latin, but at present they are given in Dutch; and, by this arrangement, the two institutions stand in closer connexion.

The following are the names of the professors, and the subjects on which they lecture:

| | |
|---|---|
| F. van der Breggen, | Pathology, Nature and Treatment of Diseases, Forensic Medicine, Pharmacology (if required). |
| W. Vrolik . . . | Anatomical Dissections, Zootomy, Physiology, Pathological Anatomy. |
| W. S. Swart . . . | Chemistry, Mathematics, Physics. |
| W. H. Vriese . . | Botany. |
| C. B. Tilanus . . | Surgery, Midwifery, Clinical Surgery. |
| G. C. B. Suringar . | Institutes of Medicine, Practice of Medicine, Clinical Medicine in St. Peter's Hospital. |

In this school, rural physicians and surgeons, (allowed to practise in both capacities, as well as in midwifery,) town surgeons, apothecaries, and midwives, are qualified. The male pupil must have attained his sixteenth year; the female, qualifying for midwife, the age of between twenty and thirty, and possess the requisite abilities and capacity, to be admitted by the Provincial Board. This admission costs the pupil three florins; in addition to which, he pays fifteen florins for every course he attends.

The surgical pupil is required to attend the following courses:

First Year:—*First Course,* Anatomy, Natural History, Chemistry; *Second Course,* Anatomy, Physiology, Botany.

Second Year:—*First Course,* Anatomy, Physiology; *Second Course,* Anatomy, Physiology, Pathology.

Third Year:—*First Course,* Materia Medica, Pathology, Surgery; *Second Course* Materia Medica, Clinical Surgery.

Fourth Year:—*First and Second Course,* Clinical Surgery.

The student who intends practising both medicine and surgery must attend the following courses:

First Year:—*First Course,* Anatomy, Natural History, Chemistry; *Second Course,* Anatomy, Physiology, Botany, Pharmacy.

Second Year:—*First Course,* Anatomy, Physiology, Pharmacy; *Second Course,* Anatomy, Physiology, Materia Medica.

Third Year:—*First Course,* Materia Medica, Pathology, Chemistry; *Second Course,* Therapeutics, Chemistry, Surgery, Clinical Medicine.

Fourth Year:—*First Course,* Therapeutics, Clinical Medicine and Surgery; *Second Course,* Clinical Medicine and Surgery.

No student is admitted to the lectures given on Midwifery till he has attended, for the space of two years, the preparatory lectures on anatomy and pathology; and no licence is granted for giving instruction in this art until the last year of study, and after the pupil has diligently attended the lectures on the state of pregnant and puerperal women and new-born children.

Students in Pharmacy are required to attend lectures on Natural History, Chemistry, and Botany, during the first and second year after their admission.

Females, studying the Obstetric Art, are required to follow the course of study indicated by the professor; but this course is always required to be in accordance with the royal regulation of the 6th of January, 1823.

The professors hold yearly a general examination of all the pupils in the Clinic, in presence of a member of the Provincial States, a member of the town administration, and one of the Board of Direction of the Hospitals. After the examination, the professors are required to forward to the Provincial Medical Board a report on the progress of each pupil.

Students at the Universities, or at the Amsterdam Athenæum, (having been admitted as *Candidates,* or taken their degree of *Doctor,*) may, if they choose, participate in the instruction given at the Clinical School: and, indeed, there are better opportunities for the acquirement of practical skill at the hospital of this place (*St. Peter's Gasthuis,*) than at the Athenæums, as the professors at the Clinic are also physicians at the hospital.

All the patients of the hospital are at the disposal of the professor, who may remove any of them, men or women, to the Clinic attached to the hospital, and allow them to be treated by the *Candidates,* agreeably to the method prescribed in his lectures. We do not, however, approve of the plan of choosing male subjects one year, and female the next. The professor of surgery selects subjects for his clinical instruction from among all the patients under his care, as well as from the pregnant women and those recently confined.

Besides the hospital of St. Peter's, there is another, called the *Pest-house* (Pesthuis), in many respects very superior to it, at a few minutes' walk from the city. Into this, as in the former, patients requiring medical or surgical aid are admitted; with the exception, however, of pregnant women. Lunatics and venereal subjects are also received into this hospital, which are wanting in the Clinic, to render the course of instruction complete. This hospital is under the direction of a physician and a surgeon: the former resides in the hospital, the latter in the city, where he attends to his private practice, but visits the hospital every morning.

If the beautiful *Workhouse* (Werkhuis), which is too expensive for this city, and might easily be dispensed with, were converted into an hospital, which might be done at a small expense, the two hospitals might be united into one useful establishment. By this arrangement, the *Pesthuis,* situated outside of the gate, might be made useful as a lunatic asylum, in which the unhappy citizens would be better attended, and a greater probability of their recovery be entertained. It is a disgrace to our country that we possess no general lunatic asylum. Those now existing can only be considered as private madhouses, generally defective, and established more for private advantage than general good.

The Botanic Garden occupies a rather extensive piece of ground in the *Slantage,* a walk within the city, adorned with gardens and garden-houses. This garden has been rendered much more effective and beautiful by the late improvements, and we hope to see it still more complete. All the students who attend, or have attended the botanical lectures, have free admission to this garden, in which the medicinal plants are classified in a separate piece of ground. This garden is also distinguished by a large and rare collection of exotics.

The Anatomical Theatre is in a very imperfect condition, and stands in general disrepute, because culprits are executed, and other public punishments, inflicted in front of it. To the want of a good anatomical theatre, we may add that of a public anatomical and physiological museum.

A few years ago, the king offered to present our city with the beautiful anatomical and physiological cabinet of Professor Reimer; but the authorities of the city declined the offer, (as we are informed,) because they had no building fit to deposit it in. This, however, we may be allowed to doubt; as, if the authorities had applied to the Provincial Medical Board, they would very probably have provided, not only a suitable place for the reception of the cabinet, but also funds for the care of so valuable a bequest.

The excellent and extensive Physiological and Pathological Museum of Professor G. Vrolik is so celebrated that we can add nothing to its praise: a detailed catalogue of its contents would be very interesting.

The Medical and Surgical Clinic is also now occupied in making a collection of pathological specimens; and, considering the shortness of the time, a very interesting collection has already been made. This school, which has been established only eleven years, reckoned, in 1836, forty-one students, exclusive of those entered a the Athenæum.

We now entertain some hope of having a new anatomical theatre, in which room might be made for the much-wanted pathological cabinet.

### II. III. DEVENTER AND FRANECKER ATHENÆUMS.

The other two Athenæums, at Deventer and Franecker, (the latter of which was formerly a university,) are of too little importance in reference to medicine to require any further notice; though the Franecker costs the country 20,000 florins a year, and the Deventer probably very little less.

The total number of students in the universities, in 1834, was as follows:—In Leyden, 761; in Utrecht, 540; in Gröningen, 296: total, 1597. The number has been steadily increasing since this period.

It must strike every reflecting reader, that the number of institutions is disproportioned to the wants of a country containing a population of only two millions and a half; for, in the first place, the expenditure of 258,000 florins (21,500*l.*) yearly for the universities alone, independently of the cost of the Athenæums and clinical schools, is too great for a small territory, already oppressed with other taxes. In the second place, the great number of institutions prevents the extension and perfection of them individually. At present, each department of a science can be superintended by only one professor, and from this will arise that want of emulation in teachers, as well as in pupils, so necessary in universities. From this circumstance must also result partial views of a subject, and blind belief in the dicta of the professor, so that the system of the teacher being known, there can be no difficulty in determining that of the pupil. The minds of the pupils would be better developed if different professors lectured on the branches of a science; the difference of opinions, particularly in medicine, would be smoothed down, and one tend to elucidate the other; excitement would be kept alive among the pupils, and a stronger desire for enquiry and reflection created; the consequence of which must be greater accuracy of judgment: besides which, the professors would endeavour to increase the number of their auditors by the improved delivery and selection of the matter of their lectures. These lectures are delivered at the universities in Latin, and at the Athenæums and clinical schools in Dutch.

### B. OF THE ORDERS AND QUALIFICATIONS OF THE MEDICAL PROFESSION.

The universities alone can confer the degree of Doctor. The individual desirous of this degree must have matriculated at the university, and have undergone the *examen philosophicum;* previously to being admitted to which, he must adduce certificates of attendance on lectures on the Greek and Latin languages, mathematics, physics, chemistry, and botany; on all which subjects he is tested at this examination. He next matriculates as a medical student, and attends the lectures on anatomy and physiology, zoology and comparative anatomy, general pathology, and pharmacy. On these points he is examined at the *examen pro gradu candidatûs;* having passed which, he receives the denomination of *Candidatus medicinæ*, a class already referred to as admissible to the clinical schools. This trial generally takes place from twelve to eighteen months after the *examen philosophicum*, and at it the bones and dried preparations are placed before the student for demonstration. He now proceeds, by attending lectures on materia medica and therapeutics, medicine, surgery, midwifery, forensic medicine, clinics and hospital practice, to qualify himself for the *examina pro gradu doctoratûs.* These are two in number: at the first, his proficiency in the above subjects is tested; at the second, he has a written question and aphorism from Hippocrates,—the former of which he has to answer, the latter to write a commentary on. This being completed to the satisfaction of the professors, he obtains the rank of *Doctorandus;* a grade analogous to the bachelor of medicine of the English universities. He may now immediately proceed to the degree of doctor, for obtaining which he has to print an inaugural dissertation, and defend it in public against the professors, or any individual who may choose to oppose him. Immediately after this he proceeds to the senate-room, takes the academic oath,* and receives his degree from one of the professors, who is termed his *Promotor.* Previously to each of these trials, he undergoes a preliminary private examination before one of the medical professors, who advises him to, or dissuades him from presenting himself for the public examination, according as he finds him possessed of the requisite qualifications or not. The examinations for M.D. are oral, (with the exceptions above mentioned,—i. e. the question and aphorism,) and in the Latin language. Practical examinations at the sick-bed are not required. The examinations of candidates for the degree of Doctor of Surgery are also held in Latin; and no operations on the dead body are required. The examination for the degree of Master or Doctor of Midwifery alone requires some proofs to be given of practical experience in that department of the art.

Few candidates take up the three degrees at the same time, as such a qualification would be of no advantage in obtaining a professorship, or other official appointment; of which latter there are almost none for medical men. Moreover, a doctor both of medicine and surgery can practise only in one capacity, under heavy penalties, and is required to make his election for practice between the two. It is remarkable that a doctor of medicine and of midwifery is not allowed to practise in both capacities, but that a doctor of surgery, being also a doctor of midwifery, may do so. As these branches cannot be separated, it would certainly be advisable to confer the degree of doctor on no candidate who could not produce testimonials of, at least, a theoretical knowledge of surgery. Government ought also to give or secure some advantages to those who should take the three degrees.

The degree of Master is conferred by the Provincial Medical Board on surgeons and on rural doctors, who are permitted to practise medicine, surgery, and midwifery; also on apothecaries, midwives, druggists, dentists, and oculists, who unfortunately flourish still in great numbers. The examinations held by the Provincial Medical Board, are not very strict. The surgeons, rural doctors, and apothecaries are re-

* The following is a copy of this oath:—"Testor Deum omni potentem (sancte promitto) me in curandis ægris, diætam aliaque remedia, quantum ingenii viribus assequar, ex artis regulis ad ægrotantium salutem et commodum commendaturum: nec prece, nec pretio aliave de causâ pharmacum noxium cuiquam propinaturum, nec gravidæ abortum procuraturum. Audita vel visa inter curandum, nisi Reipublicæ ea efferri intersit, silentio, suppressurum: in examine autem forensi ad judicem fideliter relaturum; quid actum, quid repertum sit, et de indole mali ex animi sententiâ religiose pronuntiaturum. Et in his omnibus pietati, honestati et conscientiæ integritati operam daturum. Hæc si sincere præstitero, nec sciens fefellero, felix mihi per Deum vita et ars esto. *Ita me Deus juvet!*

quired to know sufficient Latin to translate the Pharmacopœia Belgica: this forms the first part of the examination.

There is in every province one, and in large provinces two Provincial Boards, appointed by the king, consisting of doctors of medicine, surgeons, and apothecaries. Of these there are in all ten, in direct communication with the minister of the interior, receiving his directions, giving their opinion on proposed questions, and giving explanations when required. Their office is also to watch over the health of the province, to notice the epidemics and diseases that make their appearance, to enforce the regulations for medical practice, to examine the apothecaries' shops and surgical instruments in the country and towns in which there is no local Board, &c.

In towns in which more than two doctors of medicine reside, local Boards are appointed by the town administration: these are under the control of the Provincial Board, and their duty is to watch over the medical police, and examine the apothecaries' shops and the surgical instruments, and to make a yearly report to the Provincial Board on all medical occurrences that have come under their cognizance. These local Boards are, however, not authorised to punish infractions of the law, quackeries, &c., but merely to receive information of such occurrences, which are afterwards to be forwarded to the courts that have to decide on such cases. The Provincial Boards also report, every year, to the ministry whatever has occurred concerning medicine in their province, accompanied by such remarks as they may deem fit.

Doctors, who have graduated at one of the universities, are allowed to practise in any part of the kingdom; but surgeons and others, who have received their degree from the Provincial Boards, are allowed to practise only in the province in which they have been examined; and must undergo a new examination, if required, by the Board of the province in which they are desirous of practising. Dentists and oculists are excepted from this regulation, and may practise their profession in any part of the kingdom.

### MILITARY MEDICAL ESTABLISHMENT.

The military physicians receive their medical education at the Royal Hospital at Utrecht, and are admitted as pupils after they have given proofs, in a preliminary examination, that they know the Dutch, French, and the elements of the Latin languages, and have some knowledge of arithmetic, history, natural philosophy, &c. They are divided into three classes, and are maintained partly at the expense of government, and partly at their own. They are required to study four years, or until they have acquired sufficient knowledge of the sciences enumerated in the prospectus to pass the examination. Those who apply themselves diligently receive a salary; they board themselves, but are submitted to military discipline. The means of instruction are ample, and directed by teachers of the first and second class. The course of instruction is as follows:—*The first year,* they are instructed in general and particular anatomy, chemistry, languages, drawing, botany, and pharmacy. During *the second year,* instruction in the above branches is continued, with the addition of physiology, materia medica, and surgery. *The third year* is occupied with the study of general pathology and chirurgical operations; and *the fourth* with clinical instruction in medicine, surgery, &c: they may also attend lectures at the university, and must attend the Chemical and Pharmaceutical College and the Botanical Garden.

When the candidate has given proofs of sufficient knowledge of the different branches of science on which he is to be examined, he is admitted to take his examination for the rank of officer of health of the third class, and is examined on the following subjects:—theoretical and practical anatomy, physiology, medicines and their combinations, botany, the elements of chemistry, a part of pharmacy, general pathology, general treatment of the sick, the particular pathology and treatment of diseases which suddenly threaten life, systematical description of general and particular surgical diseases, and their medical treatment, bandaging, appropriate applications in cases of operations, the practice of minor operations, &c.

This examination having been satisfactorily passed, the candidate is promoted to the rank of officer of health of the third class, with a salary of 600 florins (50*l.*), which is equal to the military rank of second lieutenant; his studies are continued in the hospitals and the military corps to which he is attached, under the superintendence of the inspector-general of the medical staff.

To be promoted to the second class, which can be done only after some years' service, a new examination is necessary: this embraces the higher branches of surgical anatomy, physiology and its literature, a more extensive enquiry into simple and compound medicines, chemistry and its application, botany, antidotes as applicable in cases of poisoning; pathology, particularly with regard to military cases; surgical diseases, particularly such as have reference to soldiers; surgical instruments, operations, dissections as referring to juridical medicine. Practically, the examination comprehends a demonstration on a dead subject or a preparation; the treatment, for fourteen days, of four patients affected with acute or chronic diseases, the drawing up the *Historia Morbi*, and the performance of one or more operations on a dead or living subject. The rank of first lieutenant, and a pay of 900 florins (75*l.*), are given to officers of this class.

To be admitted to the first class, and become surgeon-major, a third examination, embracing a greater variety of subjects, must be passed; and only after a service of some years. This examination includes anatomical demonstration relating to surgical practice; the treatment of eight patients during a period of four weeks, with an analysis of the different methods of treatment. Officers of this class hold the rank of captain, and receive a salary of 1600 florins (133*l.*)

Besides these, there is one still higher rank to be obtained; namely, that of first officer of health, which gives the rank of staff-officer.

The five oldest surgeons-major receive, after they have served for ten years, a salary of 1800 florins (150*l.*); after twenty years' service, they receive 2000 florins (166*l.*) a year, and have then the same privileges as physicians and surgeons to practise in towns.

The apothecaries of the army are also divided into three classes, and at every promotion must undergo a new examination, on natural history, botany, chemistry, pharmacy, &c. The subjects on which the candidate is to be examined are named by the inspector-general, who has the privilege of being present at the examination: this is held at the Royal Hospital at Utrecht, and by the resident professors. At the head of this military medical administration is the inspector-general of the land and sea medical service, who, at this time, is M. Bernard.

### MEDICAL LITERATURE.*

Owing to its contracted territory, and its intimate connexion with other countries, of which the languages are more generally studied and more extensively known, and from which, particularly Germany and France, books are continually imported, the medical literature of Holland is at present rather circumscribed. Still it can boast of many distinguished authors, whose works are well known throughout Europe; and, among others, the distinguished friend to whose kindness we are indebted for the foregoing sketch.† Several of these works are noticed in our Lists of Books received for Review, and we have now before us a catalogue of the principal ones which have appeared in Holland since 1834: of these, twenty in number, twelve are in the Dutch language, and eight in Latin, and they appeared at the following places: at Amsterdam, 12; Leyden, 3; Gröningen, 2; Deventer, 1; Franecker, 1; Tiel, 1. Among the publications of the present year we observe, in Dutch, one on Percussion and Auscultation, and another on Lithotripsy. The following is a list of the journals on medicine and the allied sciences at present published in Holland, which, it will be observed, amount precisely to the same number as are published in Great Britain.

* By one of the Editors.

† We regret to learn, from the public prints, that the respected author of this Report is recently dead.—For the principal part of the account of Leyden in this Report, and also for the mode of examination for medical degrees, we are indebted to our friend, Dr. William Munk, a recent graduate of that learned university.

1. Practish Tydschrift voor de geneeskunde in al haren omvang, &c. &c. Verzameld door A. Moll en C. van Eldik. (Medicine.)
2. Tydschrift voor natuurlyke Geschiedenis en Physiologie. Uitgegeven door J. v. d. Hoeven en W. H. de Vriese. (Natural History and Physiology.)
3. Natuur en Scheikundig Archief. Uitgegeven door G. J. Mulder en W. Wenckebach. (Natural History and Chemistry.)
4. Nieuwe Schei-, Artsenymeng- en natuurkundige Bibliotheek. Verzameld door B. Meylink. (Chemistry, Pharmacy, &c.)
5. Hippocrates. Magazyn toegewyd aan den geheelen omvang van de geneeskunde, &c. (Medicine.)
6. Aesculap. Een Vaderlandsch Tydschrift voor theor. en pract. bydingen in het gebied der genees-, Heel-, en verloskundige wetenschappen. (Medicine, &c.)
7. Tydschrift voor genees-, Heel-, Verlos- en Scheikundige Wetenschappen. (Medicine, &c.)

---

